# Rilonacept and Anakinra in Recurrent Pericarditis: A Systematic Review and Meta-Analysis

**DOI:** 10.7759/cureus.31226

**Published:** 2022-11-08

**Authors:** Ziad R Affas, Banan Q Rasool, Sneha A Sebastian, Rafe S Affas, Sayran K Mohamadtahr, Nagham H Saoor, Aya N Mohammad, Ghada H Saoor, Bzhar A Husain, Rowaid Touza, Ghaid Touza, Shwan Amen, William Nazzaro

**Affiliations:** 1 Internal Medicine, Henry Ford Health System, Clinton Township, USA; 2 Internal Medicine, Hawler Medical University, Erbil, IRQ; 3 Research, Larkin Community Hospital, South Miami, USA; 4 Research, Henry Ford Health System, Clinton Township, USA; 5 Pharmacy, Hawler Medical University, Erbil, IRQ; 6 Internal Medicine, University of Al-Mustansiriyah College of Medicine, Mustansiriyah, IRQ; 7 Cardiac Surgery, Surgical Specialty Hospital, Erbil, IRQ; 8 Cardiology, Henry Ford Health System, Clinton Township, USA

**Keywords:** anti-interleukin 1, recurrent pericarditis, rilonacept, anakinra, pericarditis

## Abstract

Interleukin 1 (IL-1) has been indicated as a mediator of recurrent pericarditis. Rilonacept, a soluble IL-1 receptor chimeric fusion protein neutralizing interleukin 1 alpha (IL-1α) and interleukin 1 beta (IL-1β), has demonstrated promising results in a phase II study in recurrent or refractory pericarditis. Anakinra is a recombinant inhibitor of the IL-1 receptor with a demonstrated reduction in the incidence of recurrent pericarditis. Definite pharmacological management of pericarditis is key to preventing recurrences, mostly treatment options for recurrent pericarditis refractory to conventional drugs. Here we critically discuss the existing therapy options for recurrent pericarditis, with a focus on new pharmacological approaches: rilonacept and anakinra.

A systematic search was conducted across online databases such as PubMed, Cochrane, Google Scholar, ScienceDirect, CINAHL, Scopus, and Embase to obtain clinical trials that assess the effectiveness of anti-interleukin 1 therapy such as anakinra and rilonacept in the management of recurrent pericarditis. Our study concluded that anti-interleukin 1 therapy significantly improved both the quality of life and the clinical outcomes of the study population. These outcomes were most prominent with the use of rilonacept and anakinra in the trial treatment. Rilonacept and anakinra are valuable options in case of recurrent pericarditis refractory to conventional drugs.

## Introduction and background

**Figure 1:**
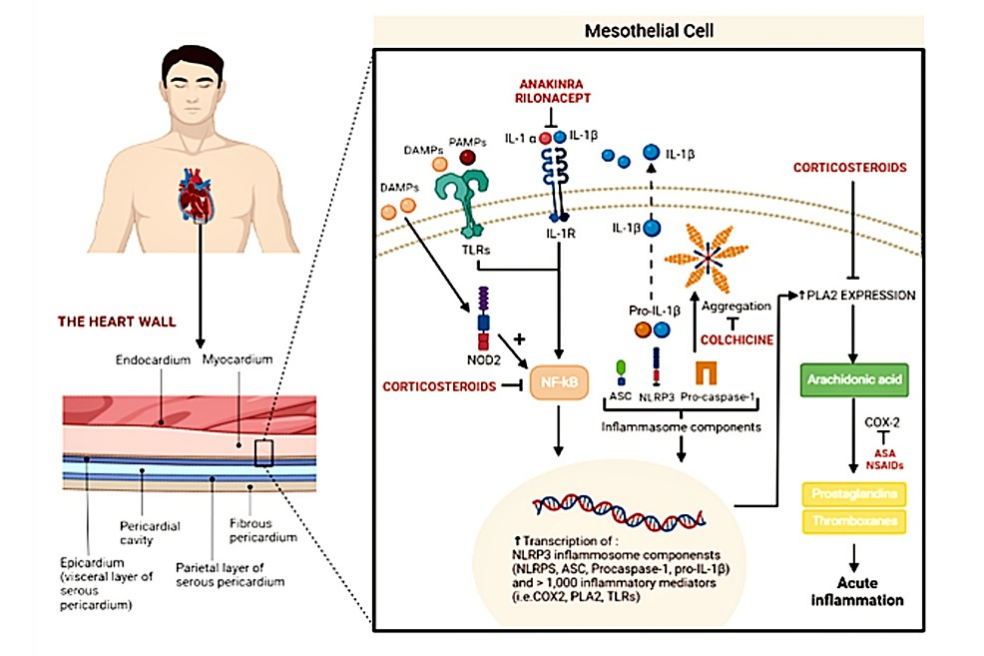
Pathophysiology of acute and recurrent pericarditis The image is created by the authors. IL-1α: Interleukin 1 alpha; IL-1β: Interleukin 1 beta; IL-1R: Interleukin 1 receptor; DAMPs: Damage-associated molecular patterns; PAMPs: Pathogen-associated molecular patterns; PLA2: Phospholipase A2; COX-2: Cyclooxygenase-2; NSAIDs: Non-steroidal anti-inflammatory drugs; ASA: Aspirin; NOD2: Nucleotide binding oligomerization domain containing 2; TLR: Toll-like receptor; NLRP3: NOD-, LRR-, and pyrin domain containing protein 3; ASC: Apoptosis-associated speck-like protein containing a CARD or PYCARD

Pericarditis is an inflammatory disease affecting the pericardial sac, resulting from a variety of stimuli that trigger a stereotyped immune response [1]. Despite a patient receiving optimum medical therapy after contracting pericarditis, an average of 23% of these patients experience a recurrence of this condition [2]. Recurrent pericarditis is a chronic condition that weakens the host and is characterized majorly by relapsing and remitting pericardial inflammation (Figure 1) [1,2]. The main symptoms include chest discomfort and shortness of breath, which eventually lead to functional limitation and a poor quality of life. Patients who develop pericarditis for a second time within four weeks of having no symptoms are diagnosed with recurrent pericarditis [2]. The criteria for diagnosis of acute pericarditis should be based on the determination of two of the following four criteria: history of chest pain, pericardial friction rub, characteristic electrocardiographic changes, and lastly emergence of a new or deteriorating existing pericardial effusion [3]. Laboratory workups such as assessment for inflammation and myocardial damage and cardiac magnetic resonance (CMR) are essential in diagnosing complicated cases [3].

Recurrent pericarditis treatment consists of an NSAID (nonsteroidal anti-inflammatory drug), typically with a 2 to 4-week taper after the resolution of symptoms, along with at least six months of colchicine (with weight-adjusted dosing) [3,4]. Corticosteroids (at low doses) should be reserved for those who have failed multiple attempts at therapy with an NSAID plus colchicine or patients with a history of autoimmune disease, pregnancy, or immune checkpoint inhibitor-associated pericarditis. Anti-IL-1 therapy (e.g., anakinra and rilonacept) is beneficial in patients with refractory, corticosteroid-dependent disease. IL-1, a pro-inflammatory cytokine that is a downstream mediator of the neutrophil-to-lymphocyte ratio family pyrin domain containing 3 (NLRP3) inflammasome signaling pathway, stimulates the synthesis of inflammatory molecules such as cyclo-oxygenase and prostaglandins. The IL-1 pathway is also an important mediator of systemic inflammation in pericarditis [4].

Rilonacept is a long-acting dimeric fusion protein developed with the cytokine trap technology and has a high affinity for both IL-1β and IL-1α [1]. Recent evidence documented that rilonacept can provide rapid and long-lasting clinical and laboratory benefits in adult patients with recurrent pericarditis (≥2 relapses) [1]. In an open-label multicenter study including adult patients with idiopathic or postpericardiotomy recurrent pericarditis with ≥2 prior pericarditis recurrences treated with rilonacept 320 mg subcutaneous (SC) weekly maintenance in a six-week base treatment period, the authors reported a rapid and sustained improvement in pain, inflammation, and health-related quality of life [1,5]. Recent research suggests that anakinra, another recombinant inhibitor of the IL-1 receptor can also reduce the recurrence of pericarditis by six times [1]. Corticosteroid-dependent and colchicine-resistant recurrent pericarditis is a challenging management problem, in which conventional anti-inflammatory therapy (NSAIDs, colchicine, corticosteroids) is unable to control the disease. Here we aim to analyze the treatment strategies for recurrent pericarditis in light of the up-to-date evidence and recommendations on anti-IL-1 agents.

## Review

Methods

Search Strategy

A search was conducted on numerous online databases including PubMed, Cochrane, Google Scholar, ScienceDirect, CINAHL, Scopus, and Embase. Keywords used to search the online databases included: "pericarditis", "recurrent pericarditis", "rilonacept", "anakinra", and "anti-interleukin 1 therapy". Our search was only limited to studies that were published from 2010-2022 and we searched until September 22, 2022. All articles included in the study were published in the English language. The study designs included in this systematic review were randomized controlled trials (RCTs), clinical trials (CTs), and retrospective cohort studies related to anti-IL-1 therapy in recurrent pericarditis. Careful analysis of the included studies was done to eliminate duplicate articles and ensure comprehensive compilation. Table 1 describes the search strategy.

**Table 1:** Search strategy RCT: Randomized controlled trial

Database	Search Terms	Limits	Total Search Results
PubMed	“Recurrent pericarditis”, “anti interleukin-1”, “anakinra”, “rilonacept”	2010-2022, RCTs & observational studies, English language	2 articles
Scopus	“Recurrent pericarditis”, “anti interleukin-1”, “anakinra”, “rilonacept”	Last 10 years, English language	1 article
ScienceDirect	“Recurrent pericarditis”, “anti interleukin-1”, “anakinra”, “rilonacept”	Last 10 years, English language	4 articles
Cochrane	“Recurrent pericarditis”, “anti interleukin-1”, “anakinra”, “rilonacept”	Last 10 years, English language	3 articles
Google Scholar	“Recurrent pericarditis”, “anti interleukin-1”, “anakinra”, “rilonacept”	Last 10 years, English language	4 articles

Inclusion and Exclusion Criteria 

Articles had to meet the following inclusion criteria: (1) any RCT and CT analyzing anti-interleukin 1 therapy in recurrent pericarditis, (2) studies that included outcomes of quality of life of patients during or after the study period, and (3) studies published in English. Exclusion Criteria were as follows: (1) all systematic reviews and pilot study discussions were excluded from the meta-analysis due to reviewer bias, (2) studies in which data did not satisfy the topic or area of study, (3) studies not published within the last two decades were excluded with a preference for those published recently and (4) studies that were not published upon the writing of this review were not considered.

Article Selection

**Figure 2:**
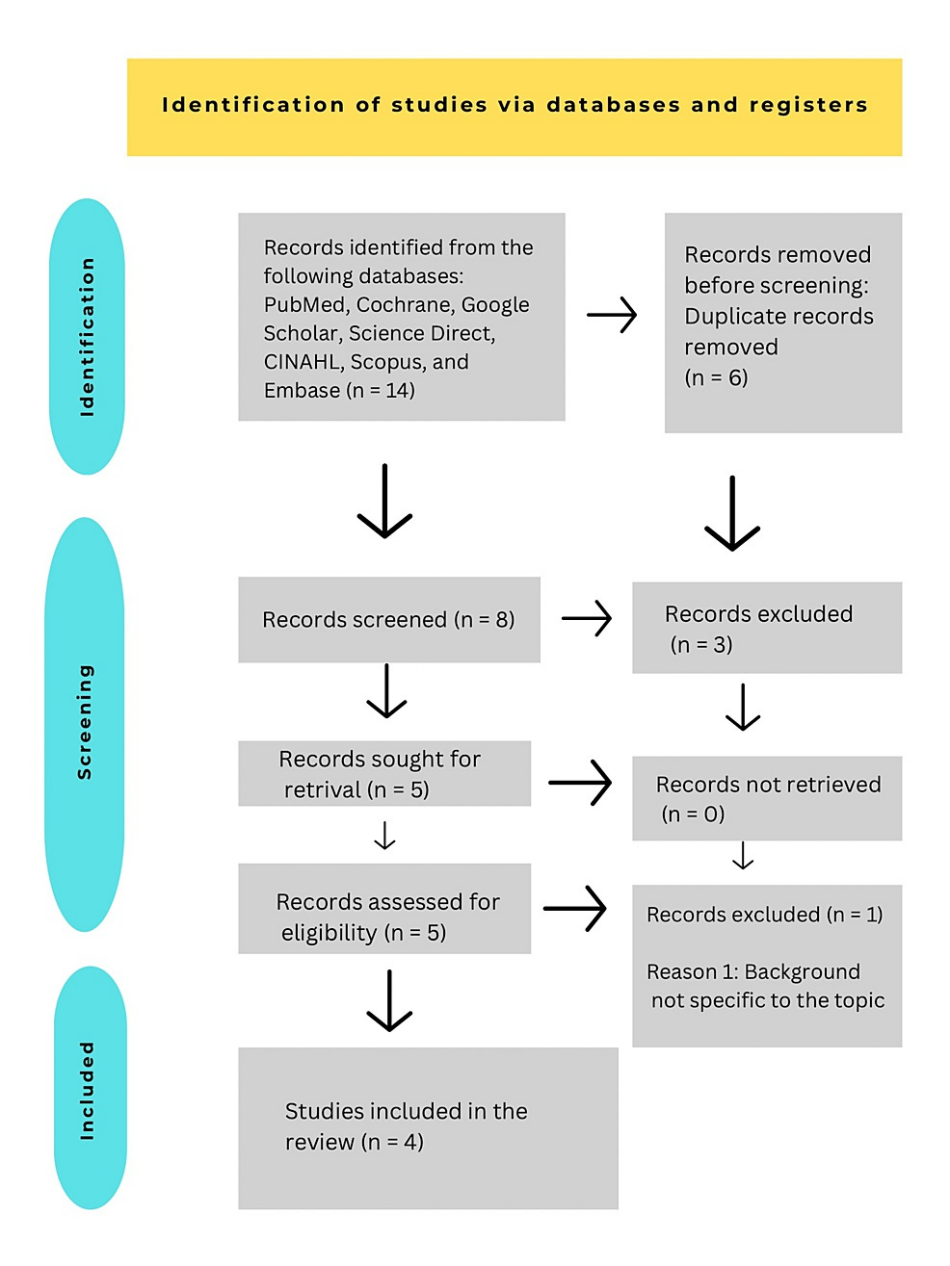
PRISMA flow diagram of study selection n = number; PRISMA: Preferred Reporting Items for Systematic Reviews and Meta-analyses

After the initial search, duplicate studies were excluded, and two reviewers (RA and SM) independently screened the articles according to the inclusion and exclusion criteria. The articles were first screened based on the title and abstract to determine their eligibility for a full-text review; any disagreement was resolved by the adjudicator (BQR). For the studies eligible for a full-text review, both reviewers screened the article for its (1) study design (2) methodology, and (3) study sample. The reference lists of included papers were screened for additional relevant references and a backward and forward citation search was also performed to make the review entirely comprehensive. This has been shown with details in the flow chart given below (Figure 2).

Quality Assessment

The selected articles were appraised for quality using preferred reporting items for systematic reviews and meta-analyses (PRISMA) checklist adequate for this particular study design. The studies were assessed for quality and included in the study.

Data Analysis

The extraction of baseline characteristics was a critical part of this systematic review and meta-analysis. Two independent researchers (SAS and SM) extracted these baseline measures to derive the most consistent and viable baseline characteristics for incorporation in the systematic review and meta-analysis. These results were then tabulated and documented in (Table 2) in the results section. Through the use of the Cochrane toolkit for systematic review and meta-analysis, odds ratios (ORs) or hazard ratios (HR) were calculated using a 95% confidence interval.

Outcome

The primary outcome of interest for this study was to determine the effectiveness of anti-IL-1 agents rilonacept and anakinra in the treatment of recurrent pericarditis. The secondary outcomes were to determine the changes in the quality of life, incidence of adverse events that required treatment discontinuation, reduction in the pericarditis-reported pain and inflammation, and other symptoms resolution.

Risk of Bias 

**Figure 3:**
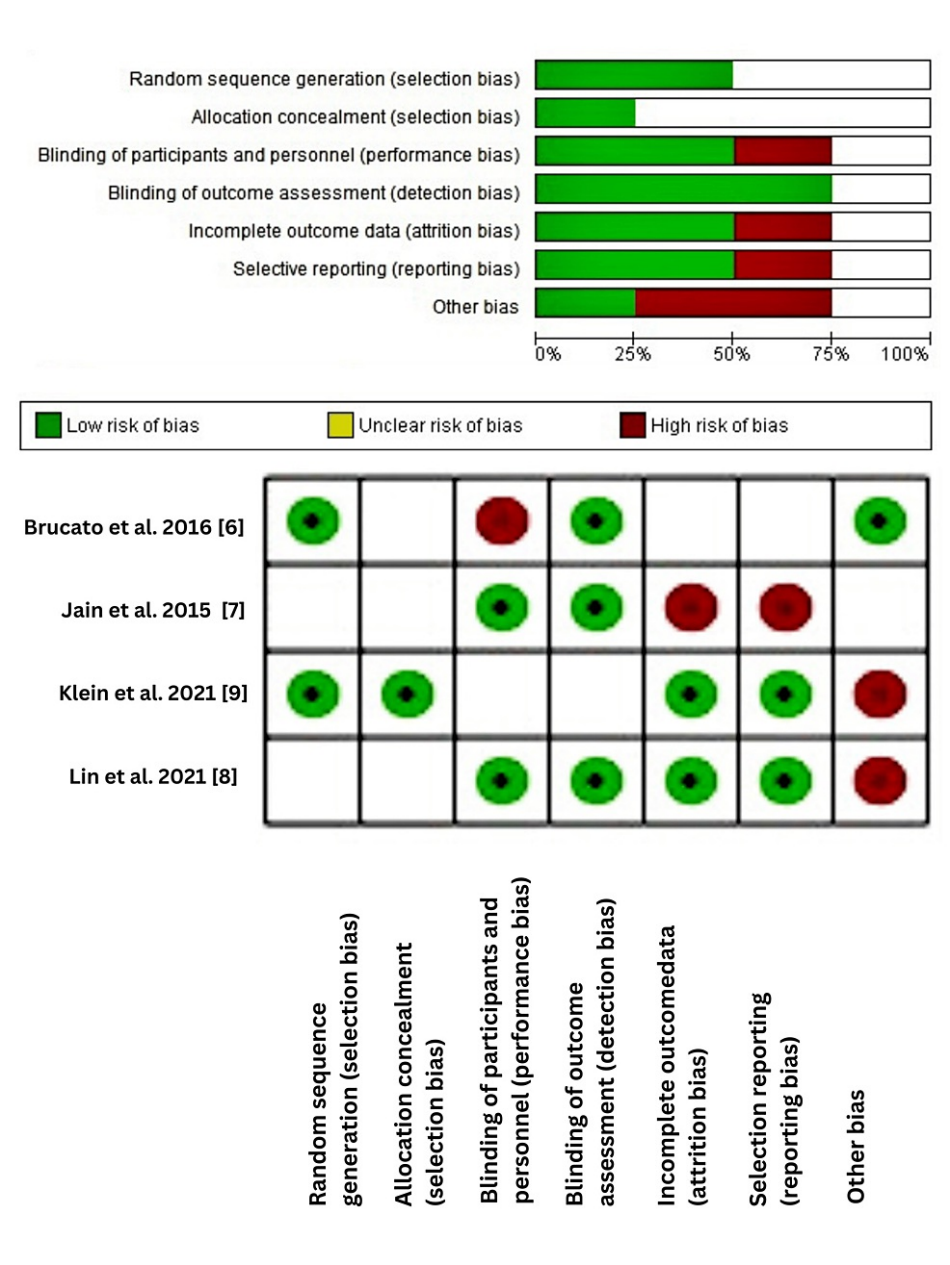
Risk of bias graph Review authors' judgments about each risk of bias item presented as percentages across all included studies and the summary of the risk of bias for each study

Measures like obscuring randomization, inclusion/exclusion criteria, individual screening, blind-ended data processing, and study considerations to treat analysis were taken into consideration to minimize the rate of bias. The healthcare population providing the treatments could not be blinded. Cochrane Handbook for Risk of Bias, as shown in Figure 3, was used to evaluate the overall risk of bias, which was determined. The risk of bias for the studies was determined as having a high, low, or unclear risk of bias. Figure 3 below represents data derived from sequence generation, allocation concealment, blinding of participants, personnel and outcome assessors, incomplete data, selective outcome reporting, and other risk areas addressed in the Cochrane toolkit summarizes the risk of bias for each study separately.

Results

Search Results

Four studies assessing the effectiveness of anakinra and rilonacept in recurrent pericarditis met all the inclusion and none of the exclusion criteria (Figure 2). Of these, two were RCTs, one CT, and one retrospective cohort study. As per the study analysis, the anti-IL-1 agents (anakinra, rilonacept) were effective in the resolution and reduction of recurrences, compared with placebo in the treatment of recurrent pericarditis. Results from a Phase II CT of rilonacept concluded that it is an effective alternative to corticosteroids for improving the quality of life in patients with recurrent pericarditis. In another retrospective study, the treatment with anakinra showed almost all participants recovering from symptoms of recurrent pericarditis. The included studies have been compiled in Table 2 with the summary of inferences and possible clinical implications.

**Table 2:** Characteristics of included studies PROMIS GH: Patient-Reported Outcomes Measurement Information System Global Health QoL: Quality of Life

No	Author and Year	Title/Focus/Topic	Drug	Number of Study Participants	Study Design	Inference
1	Brucato et al. 2016 [6]	Effect of Anakinra on Recurrent Pericarditis Among Patients with Colchicine Resistance and Corticosteroid Dependence The AIRTRIP Randomized Clinical Trial	Anakinra	21	RCT	In this preliminary study of patients with recurrent pericarditis with colchicine resistance and corticosteroid dependence, the use of anakinra compared with placebo reduced the risk of recurrence over a median of 14 months. There were 2 events in the experimental group and 9 events in the placebo.
2	Jain et al. 2015 [7]	Effectiveness and Safety of Anakinra for Management of Refractory Pericarditis	Anakinra	13	Retrospective Cohort	All patients were treated with anakinra. There was a notable 100% recovery with 12 patients fully recovering from symptoms while 1 partially recovering.
3	Lin et al. 2021 [8]	Health-related quality of life in patients with recurrent pericarditis: results from a phase II study of rilonacept	Rilonacept	25	CT	In this study Rilonacept treatment was associated with improvements in quality of life using PROMIS GH scores. Maintained/improved QoL during withdrawal of CS without recurrence suggests that rilonacept may provide an alternative to CS. Out of all the patients in the trial. Only one patient recorded serious adverse effects that required drug discontinuation.
4	Klein et al. 2021 [9]	Phase III Trial of Interleukin-1 Trap Rilonacept in Recurrent Pericarditis	Rilonacept	61	RCT	Among patients with recurrent pericarditis, rilonacept led to rapid resolution of recurrent pericarditis episodes and to a significantly lower risk of pericarditis recurrence than placebo. There were 2 events in the rilonacept group and 23 events in the control placebo group.

Assessment of results

RCT on the Efficacy of Anakinra by Brucato et al.

Researchers in this study aimed to establish whether anakinra, an IL-β recombinant receptor antagonist is effective in the treatment and management of recurrent pericarditis among patients with colchicine resistance and corticosteroid dependence [6]. The study was a double-blind, placebo-controlled, randomized open-label withdrawal trial with anakinra followed by a double-blind withdrawal step with anakinra or placebo until recurrent pericarditis occurred. The criteria for inclusion included: patients with more than two previous pericarditis recurrences, having elevated levels of C-reactive protein (CRP), being colchicine resistant, and being dependent on corticosteroids. Anakinra was administered to the whole population at a dosage of 2mg/kg per day, up to 100mg, for two months. Patients whose pericarditis resolved were then split (randomized into two groups) receiving anakinra (n=11) and placebo (n=10) for six months or until a pericarditis recurrence [6]. In this preliminary study of patients with recurrent pericarditis with colchicine resistance and corticosteroid dependence, the use of anakinra compared with placebo reduced the risk of recurrence over a median of 14 months [6].

Observational Study on the Efficacy of Anakinra by Jain et al.

This study aimed to assess the role of the recombinant interleukin-1 receptor antagonist anakinra in a series of adult patients with recurrent pericarditis as opposed to conventional therapy [7]. At the last follow-up, 11 patients (84%) had successfully discontinued concomitant NSAID, colchicine, and glucocorticoid therapy and 11 patients remained on anakinra at the end of the follow-up period. The only side effect was transient injection site reaction in four patients (31%). Researchers concluded that anakinra may be an effective alternative agent for the management of glucocorticoid-dependent recurrent pericarditis [7].

RCT on the Efficacy of Rilonacept by Lin et al. Phase II Trial

Twenty-five participants were enrolled in a multicenter, open-label, single-active-arm Phase II clinical trial of rilonacept, with an average age of 42.8 ± 10.5 years [8]. Based on their baseline symptoms and signs of pericardial inflammation, there were two groups of participants: those experiencing an active recurrence who were symptomatic with evidence of inflammation (n = 16), and those who were corticosteroids dependent but not acutely symptomatic at baseline (n = 9).

The results assessed from this study suggest a positive impact on clinical outcomes measures and improvements in quality of life when rilonacept is used in the therapy of recurrent pericarditis. Through the study period, outcomes such as pericardial pain and inflammation were assessed and significant improvements were noted [8]. Those patients that participated in the study that was initially on CS and were weaning off corticosteroids while being introduced to rilonacept reported pericardial pain with stable CRP levels, all the while reporting improved quality of life over the course of the study without recurrences. This study presented rilonacept as an alternative for treating recurrent pericarditis that not only reduced pain and inflammation of the pericardium but also reduces the risk of recurrence while improving the quality of life of the patients [8].

RCT on the Efficacy of Rilonacept by Klein et al. Phase III Trial

This was Phase III of the RHAPSODY trial. The inclusion criteria included recurrent pericarditis defined by at least two recurrences of symptoms, a pain numeric rating scale ≥4, C-reactive protein ≥1 mg/dl with concurrent use of nonsteroidal anti-inflammatory drugs (NSAIDs/colchicine/corticosteroids) [9]. The trial was a double-blind, placebo-controlled, multicenter RCT of patients with symptomatic recurrent pericarditis. The experimental group was treated with rilonacept and compared to a placebo. All the patients in the study were first subjected to 320mg of rilonacept, then followed by 160mg subcutaneously weekly for 12 weeks. Thereafter, they were randomized into an experimental group (n=30) that received 160mg subcutaneously weekly versus a placebo group (n=31) for a follow-up duration of 16 weeks.

The trial's primary efficacy endpoint was the onset of symptoms of recurrent pericarditis. The secondary endpoints were assessed at 16 weeks and included the time to normalization of the CRP level and the time by which the patients discontinued standard therapy and were receiving rilonacept monotherapy [9]. Of the total 86 patients that were enrolled, 61 patients completed the run-in period and were randomized for the trial. The study population had a mean age of 44.7 years, 57% female and 43%, male 85% of the cases were classified as idiopathic and 15% were post-cardiac injuries. The median duration of rilonacept treatment, including the run-in period, was nine months (3 to 14 months). Rilonacept initiation during the run-in period resulted in rapid resolution of the acute episode with a reduction of the pericarditis-reported pain and inflammation. All patients who were on corticosteroids at baseline were successfully tapered off and transitioned to rilonacept monotherapy within eight weeks [9].

Statistical analysis

**Figure 4:**
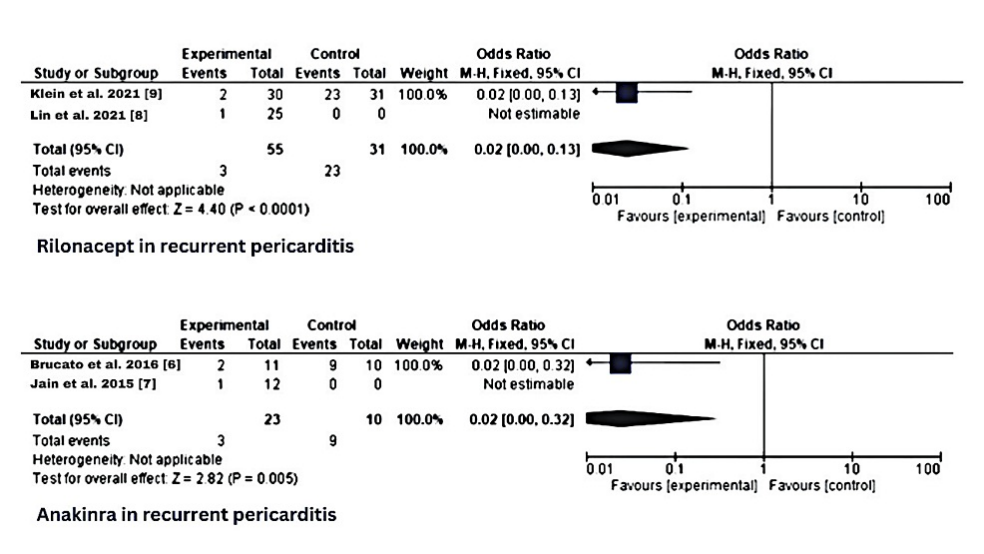
Statistical analysis of rilonacept and anakinra in recurrent pericarditis CI: Confidence interval

The forest plots below (Figure 4) indicate that each (anakinra and rilonacept) are efficient in resolving pericarditis. In the trial data obtained from both anakinra and rilonacept, the two anti-IL-1 agents showed better clinical outcomes compared to the placebo. We estimated the odds ratio of rilonacept versus placebo as 0.02 (95% confidence interval, 0.00 -0.13). And for anakinra, the odds ratio was 0.02 (95% confidence interval, 0.00 -0.32). That is, rilonacept and anakinra led to a lower risk of pericarditis recurrence than placebo. The resolution of acute episodes and the prevention of subsequent recurrences during anti-IL-1 therapy support the hypotheses that interleukin-1 is an important mediator of recurrent pericarditis in patients who have evidence of systemic inflammation.

Discussion

Recurrence is one of the most frequent complications following acute pericarditis and may occur in 30% of patients, increasing up to 50% of multiple recurrences refractory to conventional treatment [10]. Corticosteroid-dependent and colchicine-resistant recurrent pericarditis is a burdensome clinical situation, in which traditional anti-inflammatory therapy (NSAIDs, colchicine, corticosteroids) is ineffective to treat the disease. Recent advances have shown impressive results by anti-IL-1 agents in refractory recurrent pericarditis. We critically analyzed the current state of the art of therapy for recurrent pericarditis, with a focus on anti-IL-1 agents rilonacept and anakinra. As per evidence, antagonism of the IL-1 is highly effective to prevent recurrences with proven autoinflammatory features. Anakinra is the first anti-IL-1 agent (recombinant antagonist of IL-1 receptor) with well-documented efficacy and safety in adult and pediatric patients with recurrent pericarditis [11]. Studies reported that anakinra could control symptoms of recurrence after 1-2 doses with fast and safe tapering and withdrawal of corticosteroids [6,7,11]. The most commonly reported side effect of anakinra is local skin injection site reactions after 1-2 weeks of therapy. Most of these reactions are usually transient and can be treated by anti-histamines and topical corticosteroids.

Rilonacept is another anti-IL-1 agent with a soluble decoy receptor binding both IL-1α and IL-1β [8]. The Phase III clinical trial of rilonacept including 86 patients conducted in 2021 by Klein et al. documented rapid resolution of recurrent pericarditis episodes and a significantly lower risk of pericarditis recurrence than placebo [9]. Evidence suggests recurrent pericarditis can be managed with targeted rilonacept monotherapy as an alternative therapeutic option for patients with relapse [8]. The results of this Phase III clinical trial also reported that patients treated with rilonacept may be able to discontinue colchicine and glucocorticoids [9]. Injection-site reactions and upper respiratory tract infections were the most commonly reported adverse events of rilonacept therapy.

Our study has limitations, including the limited number of RCTs and CTs; however, the effect size was relatively large and significant. This is an area with limited research. Even though all CTs on rilonacept have shown significant improvement in clinical outcomes and quality of life in patients with recurrent pericarditis, more research needs to be done in larger population samples. Stratification of patients in terms of demographics and region should be demonstrated in controlled trials for inclusion and clear assessment of the impact of the therapy across different continents and demographics. Also, more prospective studies are required to assess the safety of these agents in specific populations.

## Conclusions

Phase III clinical trial has endorsed rilonacept as a highly effective treatment option in preventing the recurrence of pericarditis with potential use as a monotherapy. The experimental groups that received rilonacept showed significant improvement in clinical outcomes like symptom resolution and quality of life. There was a remarkably lesser incidence of recurrences and reduced inflammatory markers in the experimental group compared to the placebo. In addition, rilonacept produces no serious adverse drug reactions according to the trial results. In terms of anakinra, studies concluded that it is an efficacious and safe immunomodulator for glucocorticoid-dependent recurrent pericarditis. However, more prospective clinical trials with larger population samples are required to analyze the appropriate duration of therapy, the need for tapering protocols, the utility of these agents in the various stages of pericardial inflammation, and to assess its safety in specific populations.
